# Histone deacetylase 4 alters cartilage homeostasis in human osteoarthritis

**DOI:** 10.1186/1471-2474-15-438

**Published:** 2014-12-17

**Authors:** Jingwei Lu, Ye Sun, Qiting Ge, Huajian Teng, Qing Jiang

**Affiliations:** Medical School of Nanjing University, Nanjing, 210093 China; Model Animal Research Center of Nanjing University, Nanjing, 210061 China; The Center of Diagnosis and Treatment for Joint Disease, Nanjing Drum Tower Hospital Affiliated to Medical School of Nanjing University, Nanjing, 210008 China

**Keywords:** Osteoarthritis, HDAC4, Chondrocyte, Catabolism, Homeostasis

## Abstract

**Background:**

Osteoarthritis (OA) is the most common degenerative joint disorder, and a major cause of pain and disability among the elderly. Histone deacetylase 4 (HDAC4) has been shown to be a key regulator of chondrocyte hypertrophy during skeletogenesis. The aims of present study were to investigate the expression of HDAC4 in normal and OA cartilage and its potential roles during OA pathogenesis.

**Methods:**

The knee cartilage specimen (a total of 18, 12 female and 6 male) were obtained from primary OA patients undergoing total knee arthroplasty (TKA) and normal donors. By using immunohistochemistry staining, we detected the expression patterns of HDAC4 in OA cartilage and normal cartilage respectively. To assess the potential roles of HDAC4, HDAC4 expression in human chondrosarcoma cells (SW1353) was down-regulated by transfecting small interference RNA (siRNA), thereafter, cells were treated with IL-1β or TNF-α, and the expressions of several matrix-degrading enzymes and anabolic factors were examined by using quantitative PCR.

**Results:**

The expression of HDAC4 was observed in the OA cartilage, whereas it was barely detected in the normal cartilage. The extent of HDAC4 expression had a statistically negative correlation with OA severity. We further explored that the reduction of HDAC4 level led to a significant repression of proinflammation cytokines induced up-regulated expressions of matrix-degrading enzymes (MMP1 (Matrix metalloproteinase 1), MMP3 (Matrix metalloproteinase 3) , MMP13 (Matrix metalloproteinase 13), ADAMTS4 (aggrecanase 1) and ADAMTS5 (aggrecanase 2)) in SW1353 in vitro. Moreover, knockdown of HDAC4 inhibited the expression of some anabolic genes (such as aggrecan).

**Conclusions:**

In this study, our findings suggest that the abnormal expression of HDAC4 in osteoarthritic cartilage might be implicated in promoting catabolic activity of chondrocyte, which is associated with OA pathogenesis. Thus, our findings give a new insight into the mechanism of articular cartilage damage, and indicate that HDAC4 might be a potential target for the therapeutic interventions of OA.

**Electronic supplementary material:**

The online version of this article (doi:10.1186/1471-2474-15-438) contains supplementary material, which is available to authorized users.

## Background

Osteoarthritis (OA) is the most common joint disorder and a major cause of pain and disability in the elderly, which is characterized by degradation of cartilage, narrowing of joint space, osteophytes formation and remodeling of subchondral bone. A variety of risk factors have been recognized to contribute to the pathogenesis of OA, such as age, sex, primary injury, genetic predisposition, and mechanical factors. However, no effective disease-modifying treatment approach for OA has been developed to date. With an accumulating number of researches into this disease, epigenetic factors have been implicated in the pathogenesis of OA in recent years [1]. Epigenetics means descendible phenotype changes without changes of DNA sequences [2]. Two of the most characterized epigenetic modifications are DNA methylation and post-translational modification of histones. As one of the important mechanisms of epigenetics, acetylating modification of histone is controlled by histone deacetylases (HDACs). It is believed that loss of acetylation could compact the chromatin structure, leading to the transcriptional repression of specific genes [3].

HDACs fall into two families: the zinc-dependent classical HDACs family, and the SIR2 family of NAD^+^-dependent HDACs. The classical HDACs family has been divided into three classes [4]: Class I HDACs (HDAC1, 2, 3 and 8) show homology to yeast RPD3 gene, while Class II HDACs (HDAC4, 5, 6, 7, 9 and 10) are more related to yeast HDA1 gene [5]. HDAC11 alone represents class IV HDACs [4].

HDAC4 has been identified to have a crucial role in the regulation of chondrocyte hypertrophy during skeletogenesis in mice. HDAC4-null mice displayed aberrant chondrocyte hypertrophy and subsequent premature ossification in the chondrocostal cartilage [6]. Interestingly, Trichostatin A (TSA) and sodium butyrate, acting as global inhibitors of class I/II HDAC, have been shown to inhibit metalloproteinase gene expression in chondrocytes and block cartilage resorption [7]. However, the specific HDACs involved in OA, still remain to be uncovered.

Here, we investigated the potential roles of HDAC4 in OA pathogenesis and effects of HDAC4 on cartilage catabolism and anabolism. We found a statistically negative relationship between HDAC4 expression level and severity of OA. What’s more, the down-regulating of HDAC4 is associated with the decrease of cytokine-induced matrix catabolic genes expression. The results suggested HDAC4 may be involved in the pathogenesis of OA development and probably be a potential target in OA treatment strategy.

## Methods

### Human cartilage sampling

Tissue samples of knee cartilage (removed from femoral condyle and tibial plateau) were obtained from a total of 18 donors: 17 OA donors ranging in age from 46 years to 82 years (5 male and 12 female) and 1 normal donor in the age of 34 years (male). Full thickness cartilage samples were harvested. All samples were graded according to a modified Mankin score criteria [8] (Table 1), for which 0—3 points represented normal and ≥4 points meant OA. Osteoarthritic cartilage was collected from patients undergoing total knee arthroplasty (TKA). Normal cartilage was harvested under the approval from ethics committees of Medical School of Nanjing University, and all individuals undergoing surgery were provided with full written informed consent before the operative procedure.

**Table 1:** **Modified mankin scoring criteria**

	Score
**Structure**	
Normal	0
Surface irregularities	1
Pannus and surface irregularities	2
Clefts to middle zone	3
Clefts to deep zone	4
Clefts to calcified zone	5
Complete disorganization	6
**Cells**	
Normal	0
Diffuse hypercellularity	1
Cluster/cloning	2
hypocellularity	3
**Sarafin O staining**	
Normal	0
Slight reduction	1
Moderate reduction	2
Severe reduction	3
No dye noted	4

### Histology and immunohistochemistry

Cartilage tissues were fixed in 10% formalin immediately after prosecuted in surgery. After that, all the cartilage samples underwent decalcification with 15% EDTA solution for 3 weeks. Thereafter, Dehydration, clearing and wax immersion was done, followed by paraffin embedding. 5-μm thick sections were made. Hematoxylin and eosin/safranin O staining was used to grade samples for characteristics of OA cartilage pathology according to a modified Mankin score criteria (Table 1) comprising abnormalities of structure, cellularity variations and glycosaminoglycan distribution and loss. Each sample was scored three times and the mean of the three scores was used in any analysis done.

Indirect immunohistochemical staining was applied to examine expression of HDAC4. Paraffin-fixed samples were first deparaffinized in xylene and ethanol before rehydration in water. Next, sections were incubated in trypsin for 30 min at 37°C and then water bath heated in citrate sodium for 10 min. Following a wash with phosphate-buffered saline (PBS), sections were blocked with 0.5% bovine serum albumin (diluted in PBS) for 30 min at room temperature. HDAC4 antibody (1:200 dilution; 200 μg/ml; Santa Cruz Biotechnologies, catalog no.sc-11418, Santa Cruz, CA, USA) and 0.5% bovine serum albumin as a negative control were applied and incubated overnight at 4°C. After rinsing with PBS, sections were incubated with biotinylated goat anti-rabbit secondary antibody for 1 h (1:500 dilution; Vector Laboratories Inc., Burlingame, CA, USA) and then incubated with Vectastain ABC kit (PK-6100; Vector Laboratories Inc., Burlingame, CA, USA) for 1 h at room temperature. Finally, sections were stained with a peroxidase substrate kit (DAB SK-4100; Vector Laboratories Inc., Burlingame, CA, USA) and counterstained with hematoxylin.

### Cell culture

Sw1353 human chondrosarcoma cells were grown to confluence in Dulbecco’s-modified Eagle’s medium (DMEM) supplemented with 10% fetal calf serum (FCS), 100 units/ml penicillin, and 100 μg/ml streptomycin. Cells were maintained at 37°C in an atmosphere of 5% CO_2_. SW1353 human chondrosarcoma cells have been widely used in researches as a substitute for chondrocytes. Since siHDAC4 effect was not enough in human chondrocytes, we used SW1353 human chondrosarcoma cells for this assay.

### Small interfering RNA (siRNA)

Knockdown experiments were carried out on SW1353 human chondrosarcoma cells transfected with 10 μM siHDAC4 (Santa Cruz Biotechnologies, catalog no.sc-35540, Santa Cruz, CA, USA) using Lipofectamine 2000 (Invitrogen) for 48 h, following the manufacturer’s instructions. Then incubation with or without 10 ng/mL IL-1 or 10 ng/mL TNF-α for 6 h was done before cell collection.

### Quantitative real time polymerase chain reaction

Total RNA was isolated from sw1353 cells using TRIzol. First-strand cDNA was synthesized with Takara System according to manufacturer’s protocol (Takara). RNA was quantified spectrophotometrically based on A260 using ND-1000 spectrophotometer (NanoDrop Technologies, Wilmington, DE, USA). Complementary DNA was produced using a SuperScript First-Strand Synthesis System kit (Invitrogen). Messenger RNA expression of MMP1, MMP3, MMP13, ADAMTS4, ADAMTS5, COL2A1 and aggrecan was detected by real-time RT-PCR with SYBR green detection using the ABI stepone plus real-time PCR system (Applied Biosystems). The expression levels of genes were defined from the threshold cycle (Ct) and relative values were calculated by the 2^-ΔΔCt^ method after normalizing expression to beta-actin. Gene specific primer sequences are shown in Table 2.

**Table 2:** **Primer sequences for RT-PCR analysis**

Gene	Primer
MMP1	Forward: 5′-AAAATTACACGCCAGATTTGCC-3′
	Reverse: 5′-GGTGTGACATTACTCCAGAGTTG-3′
MMP3	Forward: 5′-AGTCTTCCAATCCTACTGTTGCT-3′
	Reverse: 5′-TCCCCGTCACCTCCAATCC-3′
MMP13	Forward: 5′-ACTGAGAGGCTCCGAGAAATG-3′
	Reverse: 5′-GAACCCCGCATCTTGGCTT-3′
ADAMTS4	Forward: 5′-GAGGAGGAGATCGTGTTTCCA-3′
	Reverse: 5′-CCAGCTCTAGTAGCAGCGTC-3′
ADAMTS5	Forward: 5′-GAACATCGACCAACTCTACTCCG-3′
	Reverse: 5′-CAATGCCCACCGAACCATCT-3′
Aggrecan	Forward: 5′-ACTCTGGGTTTTCGTGACTCT-3′
	Reverse: 5′-ACACTCAGCGAGTTGTCATGG-3′
COL2A1	Forward: 5′-TGGACGATCAGGCGAAACC-3′
	Reverse: 5′-GCTGCGGATGCTCTCAATCT-3′
Beta-Actin	Forward: 5′-CATGTACGTTGCTATCCAGGC-3′
	Reverse: 5′-CTCCTTAATGTCACGCACGAT-3′

### Western blotting

Cells were obtained and solubilized at 4°C with lysis buffer (40 mM Tris–HCl, 150 mM NaCl, 0.5% Sodium Deoxycholate, 1% Nonidet P40, 0.1% SDS, and protease inhibitors), incubated on ice for 30 min and centrifuged at 12,000 rpm for 20 min at 4°C. Protein concentrations in lysates were determined by the BCA (bicinchoninic acid) protein assay kit (Pierce) with bovine serum albumin as the standard. Thirty micrograms of protein extract were resolved on a 8% SDS-polyacrylamide gel, as required and then transferred onto nitrocellulose blotting membrane (Pall). The membranes were probed with appropriate primary antibodies and detected using peroxidase-conjugated anti-rabbit antibodies (1:5000) and visualized by ECL (Pierce). The primary antibodies used were rabbit polyclonal anti-HDAC4 (1:500) from Santa Cruz Biotechnology.

### Quantification of HDAC4 in cartilage

To assess expression and distribution of HDAC4, we counted positive and negative cells from cartilage superficial zone to the deep zone (using a Olympus BX53F Microscope at 200× magnification). We identified superficial zone (SZ), middle zone (MZ) and deep zone (DZ) according to variations in cell morphology, cell density, cell metabolism, and the pericellular matrix (PCM). Chondrocytes in the SZ were characterized by their elongated, flattened shape, parallel orientation to the cartilage surface and their lack of PCM. SZ comprises the first 10–20% of full-thickness articular cartilage. The MZ spans the next 40–60% of cartilage thickness and chondrocytes in this zone were of rounded shape and with an randomly organized orientation relative to the surface. DZ contains ellipsoid chondrocytes with an extensive PCM and an organization of 3 or more cells in groups. The counting was repeated 3 times for each section. The frequency of positive cells was displayed as a percentage relative to the total number of cells counted in each zone.

### Statistical analysis

Linear regression analysis and Pearson’s correlation were used to assess the relationship between the severity of OA and the extent of HDAC4 expression. Unpaired t tests were performed to determine the statistically significant differences between two groups. All statistical analyses were performed with GraphPad Prism (version 5.0; GraphPad Software, Inc., San Diego, CA).

## Results

### HDAC 4 expression is upregulated in OA cartilage

**Figure 1:**
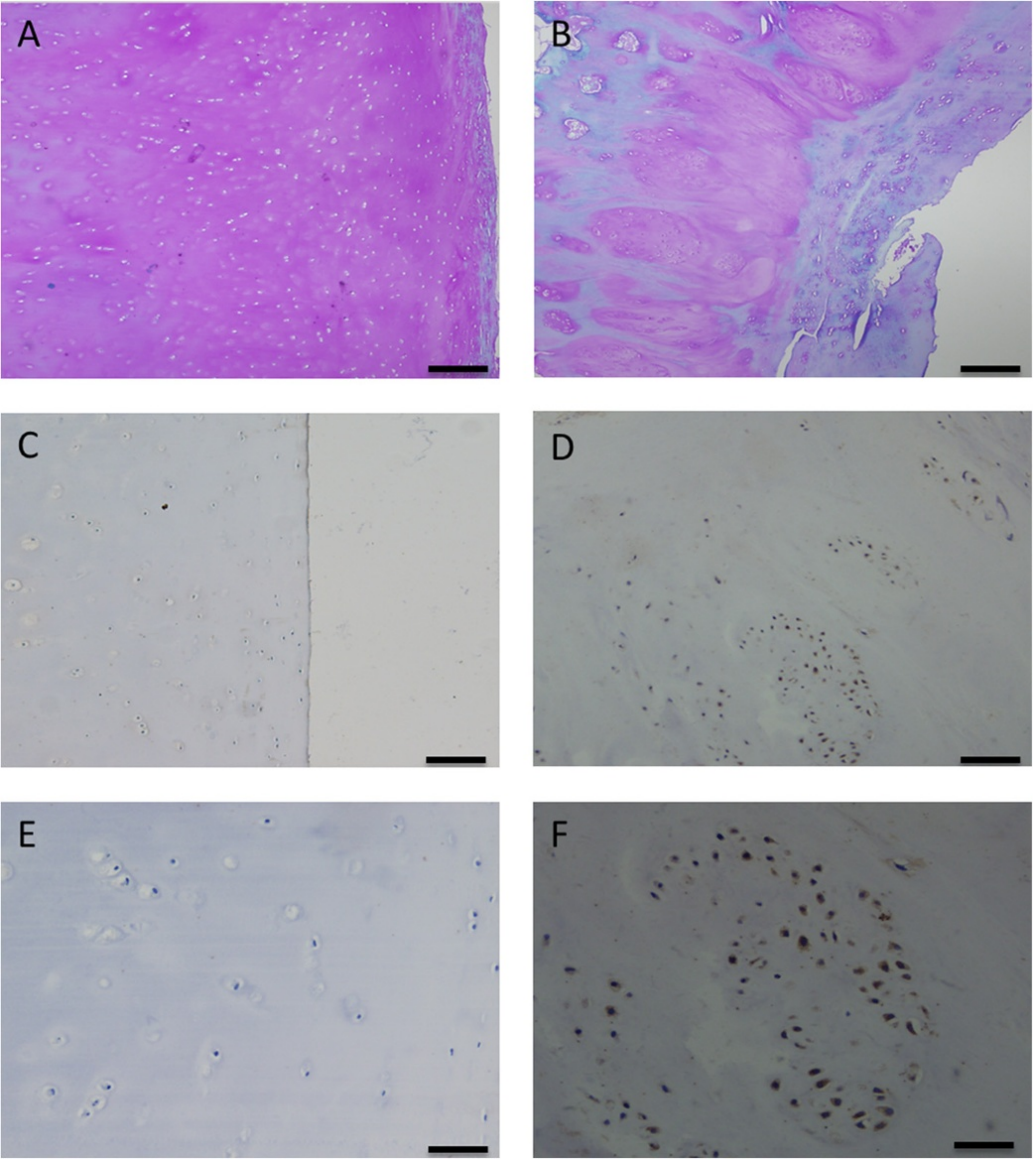
**Localization of HDAC4 in OA cartilage and normal cartilage.** Aggrecan loss was much more severe in OA **(B)** than in normal cartilage **(A)**. HDAC4 had significantly higher expression in OA **(D,F)** than in normal cartilage **(C,E)**. **A**, **B**, **C** and **D**: 100x magnification (scale bar 100 μm); **E**,**F**: 200x magnification (scale bar 50 μm). Immunoreactive products are stained brown.

**Figure 2:**
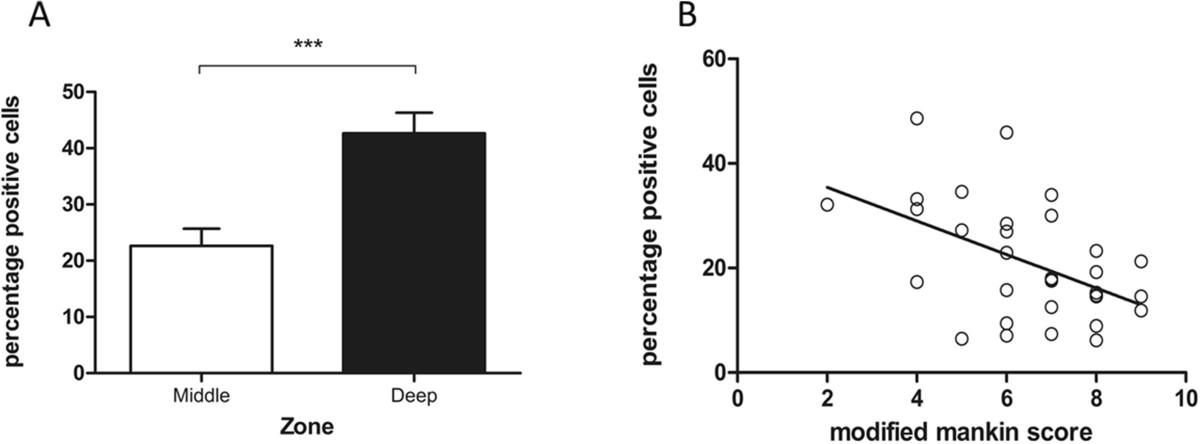
**Statistical analyses of immunohistochemistry staining. (A)** Zonal variation in HDAC4 expression in OA cartilage. The results are expressed as mean ± SEM. (n = 17; ***P < 0.001) **(B)** Overall relationship between Mankin score and expression of HDAC4. The extent of HDAC4 expression decreases linearly with increasing severity of OA (n = 34, r = −0.49, P = 0.0041).

To investigate the role of HDAC4 in OA pathogenesis, we initially detected expression level of HDAC4 in human OA cartilage. Immunohistochemical study showed a significant upregulation of HDAC4 expression in OA cartilage (Figure 1C, D, E and F). In OA cartilage, HDAC4-positive cells mainly distributed in the middle zone and deep zone, especially in chondrocyte clusters (Figure 1D, F). Confirmed with the quantitative analysis of zonal distribution of HDAC4-positive cells in 17 OA donors, significantly more positive cells were located in deep zone than in middle zone (Figure 2A), whereas HDAC4-positive cells were hardly observed in superficial zone.

### HDAC4 expression decreases with increasing OA severity

There was a significant negative correlation between ‘modified’ Mankin score and HDAC4 expression determined by quantification analysis of immunohistochemistry. Overall, the extent of HDAC4 expression was found to decrease linearly with the increasing severity of OA (Figure 2B) (r = −0.49, P = 0.0041). This relationship was consistent and significant both in femoral condyle (r = −0.54, P = 0.0265) and tibial plateau (r = −0.53, P = 0.0366).

### Inhibition of HDAC4 attenuates induction of catabolic genes expression in chondrocytes

**Figure 3:**
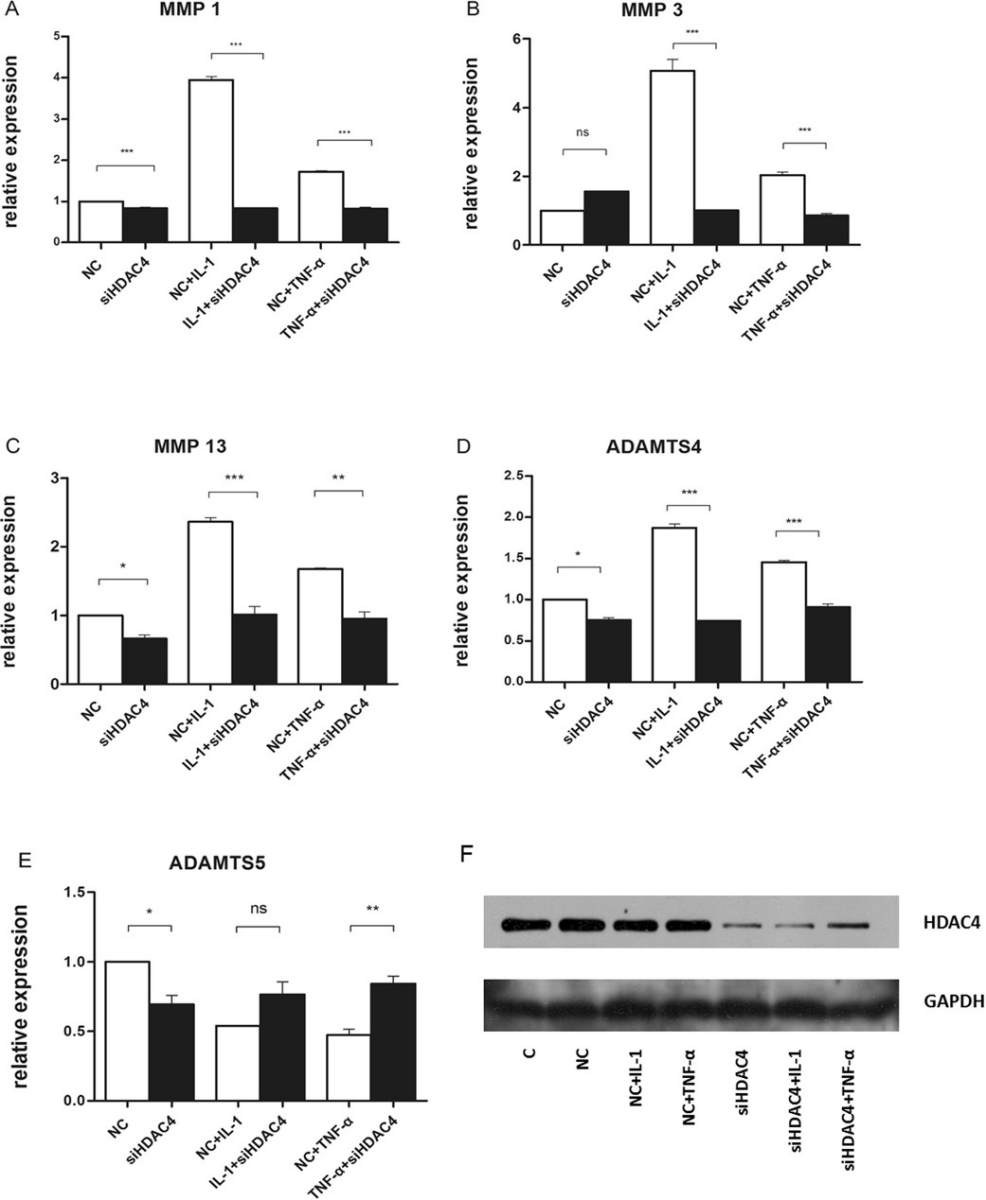
**Relative expression of key catabolic genes in the absence or presence of siHDAC4.** Messenger RNA levels were quantified by real time RT-PCR and normalized to beta-actin. The expression levels of genes **(A, B, C, D and E)** were defined from the threshold cycle (Ct) and relative values were calculated by the 2^-ΔΔCt^ method. Western blot analyses **(F)** were used to compare the relative levels of HDAC4 protein with and without the treatment of siRNA ( C = control, NC = negative control). Data are presented as mean ± SEM(n = 3, in duplicate). **P < 0.01 ,***P < 0.001.

The upregulation of HDAC4 in OA cartilage suggests the possible association of HDAC4 with OA pathogenesis. This led us to investigate the effect of HDAC4 on cartilage metabolism by *in vitro* experiments. A defining feature of OA chondrocytes is their increased production of matrix-degrading enzymes such as MMPs and ADAMTSs. We therefore asked whether HDAC4 has effect on matrix-degrading enzymes expression. Sw1353 cells were treated with specific siRNA to block the expression of HDAC4. In our assay, we knocked down HDAC4 expression level to less than 30% (Figure 3F). Treatment with specific siRNA led to a significant downregulation of HDAC4 expression and treatment with IL-1 or TNF-α didn’t show much effect on the expression of HDAC4 (Figure 3F). Cartilage-specific genes expression was measured by quantitative real time polymerase chain reaction. Expression of MMP1, MMP3, MMP13, and ADAMTS4 was significantly upregulated after treatment of chondrocytes with pro-inflammation cytokines IL-1 or TNF-α (Figure 3). Indeed, upregulation of these enzymes by pro-inflammation cytokines was effectively blocked by knockdown of HDAC4 with specific siRNA (Figure 3). Among the HDAC4-regulated matrix-degrading enzymes, MMP3, MMP13 and ADAMTS4 are crucial enzymes of OA cartilage destruction. These data cumulatively demonstrate that HDAC4 promotes catabolic activity in chondrocytes by upregulating matrix-degrading enzymes. However, knockdown of HDAC4 upregulated ADAMTS5 expression in the cytokine-induced condition (Figure 3E), indicating the complexity of underlying mechanism in the regulation of OA metabolism.

**Figure 4:**
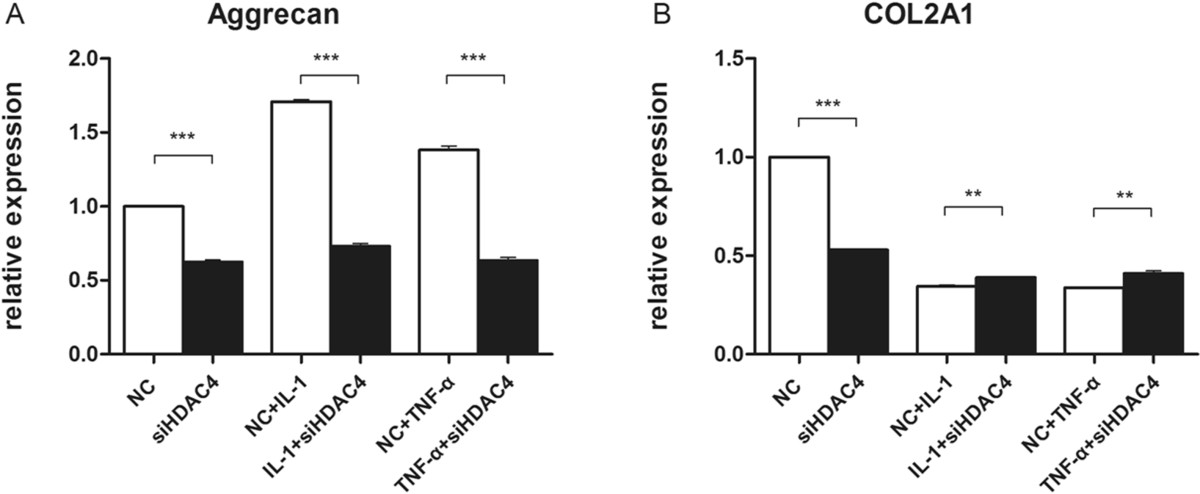
**Relative gene expression of Aggrecan and COL2A1 in the absence or presence of siHDAC4.** Messenger RNA levels were quantified by real time RT-PCR and normalized to beta-actin. The expression levels of genes **(A and B)** were defined from the threshold cycle (Ct) and relative values were calculated by the 2^-ΔΔCt^ method. Data are presented as mean ± SEM(n = 3, in duplicate). **P < 0.01 ,***P < 0.001.

In contrast to its effects on matrix-degrading enzymes, suppression of HDAC4 decreased expression levels of both aggrecan (Figure 4A) and COL2A1 (Figure 4B) without the presence of cytokines. In cytokine-induced condition, HDAC4 inhibition significantly downregulated aggrecan expression (Figure 4A), while promoted COL2A1 expression, although the extent was not large (Figure 4B).

## Discussion

HDAC4 has been identified as a key regulator of chondrocyte hypertrophy during skeletogenesis [6]. This fact drove us to investigate the possible role HDAC may have in the pathogenesis of OA, for chondrocyte hypertrophy is an essential step in the process of endochondral ossification [9], which has an important role in both cartilage degradation and osteophytes formation. In the central part of articular cartilage, where endochondral ossification remains incomplete due to lack of vascularity, proteinases cause cartilage degradation, whilst, at the periphery of articular cartilage, sufficient vascularity makes the process of endochondral ossification complete and thus osteophytes form [10].

In the present study, we observed that HDAC4 expression level was significantly higher in OA cartilage than in normal cartilage by immunohistochemical staining. The extent of HDAC4 expression had a statistically negative correlation with the severity of OA. Thus, our findings give HDAC4 a link to OA, and indicate that HDAC4 may mainly contribute to the early stage OA, in which the cartilage catabolism is more active than the end stage OA . It is well documented that pro-inflammation cytokines, such as IL-1 and TNF-α, are able to promote the chondrocytes to produce and secrete catabolic enzymes which aggravate collagen breakdown and aggrecan degradation [11]. Among them, MMP1, MMP3, MMP13 and ADAMTS4 play crucial roles in collagen degradation and aggrecan loss [12–17]. We further explored that knockdown of HDAC4 by specific siRNA in chondrocytes significantly suppressed proinflammation cytokines-mediated upregulation of matrix-degrading enzymes. These findings indicate that HDAC4 might be involved in the pathological process of OA.

Additionally, in our study, an especially strong HDAC4 immunostaing was observed in chondrocyte clusters and in the deep zonal cartilage near the tidemark. Both chondrocyte clusters and tidemark advancement are characteristic phenotypes of OA [18, 19], moreover, chondrocyte clusters show features of cell hypertrophy [20], and tidemark advancement may be partly due to the release of pro-angiogenic factors from hypertrophic chondrocytes in the deep zonal articular cartilage [21]. Together with the fact that HDAC4 controls chondrocyte hypertrophy in chondrogenesis, it will be interesting to investigate the possible correlation of HDAC4 with these two phenotypic changes.

The failure of extracellular matrix (ECM) mediated by matrix-degrading enzymes is the hallmark of OA. However, therapeutic approaches directly targeting matrix-degrading enzymes have been proved not successful in hauling the development of this pathological change. Clinical trials applying MMP inhibitors as a disease-modifying treatment have been proved unsuccessful due to severe side effects and inefficiency of inhibiting MMPs activity [22]. Our results suggest that specific inhibition of HDAC4 may be an effective way to inhibit proteinase expression. Another member from class II HDACs, HDAC7 has been implicated to promote OA development by upregulating MMP13 expression [23]. In the context of early embryogenesis, HDAC7 is specifically expressed in the vascular endothelium and maintains vascular integrity by myocyte enhancer factor-2 (MEF2) mediated inhibition of MMP10 expression [24]. A recent report [25] demonstrated that HDAC3, a class I HDAC, regulated chondrocyte hypertrophy and matrix content by inhibiting protein phosphatase Phlpp1 expression and promoting Akt activity. Chondrocytes lacking HDAC3 entered the hypertrophy stage sooner. Moreover, HDAC3-deficient chondrocytes have lower extracellular matrix production and smaller sizes than normal chondrocytes [25].

Much work has been done with HDAC inhibitors (HDACi) to infer the role HDACs serve in chondrocytes, and Trichostatin A (TSA) probably was the most frequently applied. TSA blocked proteoglycan release [26] and cartilage resorption [7] in cartilage explants, suggesting the crucial roles of HDACs in the catabolism of cartilage. TSA can also inhibit cytokine-induced metalloproteinases in chondrocytes [7, 27]. TSA may therefore be of therapeutic benefit in OA, and intra-articular injection of TSA into rabbits with experimental OA alleviated the extent of cartilage erosion, concomitant with reduced expression of IL-1 and matrix-degrading enzymes [28]. TSA also showed promise in rheumatoid arthritis (RA) models. A daily injection of TSA for 2 weeks ameliorated synovial inflammation and cartilage destruction in collagen antibody-induced arthritis (CAIA) mice [29]. The data in the study indicate that TSA might directly regulate collagenase expression in chondrocytes. In addition to broad spectrum HDACis like TSA, some isoform-selective HDACis are utilized to investigate the role of HDACs in chondrocytes. HDAC1 and HDAC2, both class I HDACs, are increased in OA chondrocytes and overexpression of them suppresses transcription of cartilage anabolic genes such as ACAN and COL2A1 [30]. Another member of class I HDACs, HDAC3, has recently been identified as a regulator of chondrocyte hypertrophy and cartilage regeneration [25]. A recent study [31] revealed that the specific inhibition of class I HDACs, particularly that of HDAC1, HDAC2, and HDAC3, showed chondroprotective effect via suppressing cytokine-induced MMP expression, showing promising utilization of isoform-specific HDACis in OA treatment strategy. With the increasing knowledge of the structures of HDAC4 and HDAC7, more specific inhibitors of these individual isoforms could soon be expected [32, 33].

HDACis were also implicated in regulating the expression of ECM components, and the mechanism appears to be complex. Short-term treatment of chondrocytes (<24 hours) with HDACis promotes anabolic gene expression (e.g., *COL2A1*, *COL9A1*, *COMP*, and *ACAN*) [30, 34], however, extended treatment suppresses many of the same transcripts [27, 30, 35]. The early positive effects may be the direct consequence of HDAC inhibition, for the overexpression of HDAC1 and HDAC2 represses the expression of *ACAN* and *COL2A1*[30], while the long-term blockade may be due to the elevation of inhibitory factors such as Wnt-5A [35] or NAB1 [36].

## Conclusions

In summary, our findings support the notion that HDAC4 expression is upregulated in human OA cartilage, and inhibition of HDAC4 might be of benefit to prevent cartilage destruction *in vitro* by suppressing matrix-degrading enzymes expression. These results implicate that HDAC4 is a potential pharmaceutical target in the inhibition of cartilage destruction. It is notable that suppression of both anabolic and catabolic activities may in fact decelerate cartilage turnover and thus preserve existing cartilage. Further studies are needed to assess whether the *in vitro* effects still remain when translated to *in vivo* models to decelerate the progression of cartilage destruction in OA.

## Authors’ original submitted files for images

Below are the links to the authors’ original submitted files for images. Authors’ original file for figure 1 Authors’ original file for figure 2 Authors’ original file for figure 3 Authors’ original file for figure 4
