# Supplementary figures and images for: Histone deacetylase 4 alters cartilage homeostasis in human osteoarthritis
Source: BMC Musculoskelet Disord. 2014 Dec 17;15:438. doi: 10.1186/1471-2474-15-438 (PMC4300609; doi:10.1186/1471-2474-15-438)

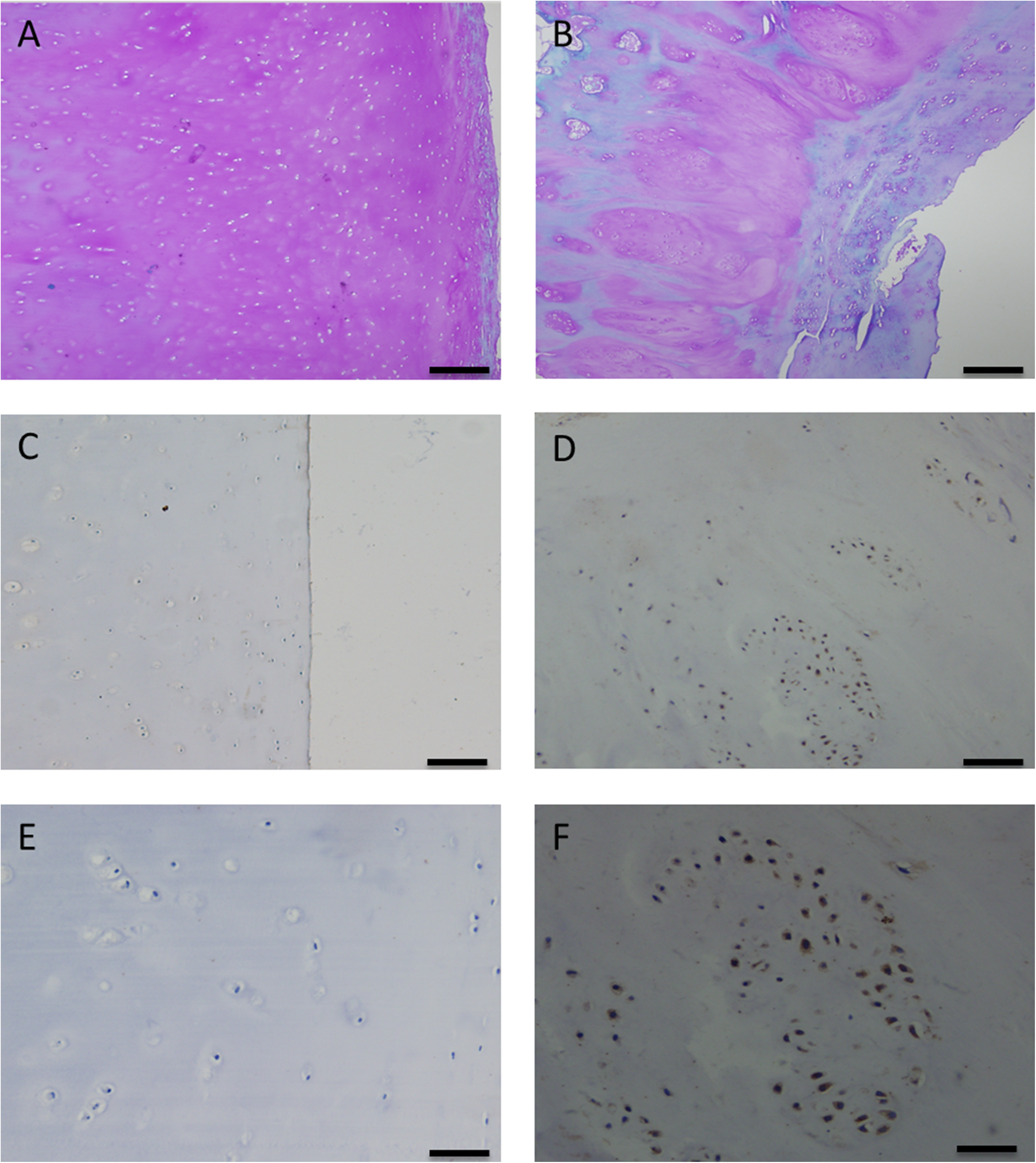

Supplement: Supplementary file 1 — Authors’ original file for figure 1 [file 12891_2014_2379_MOESM1_ESM.tif]

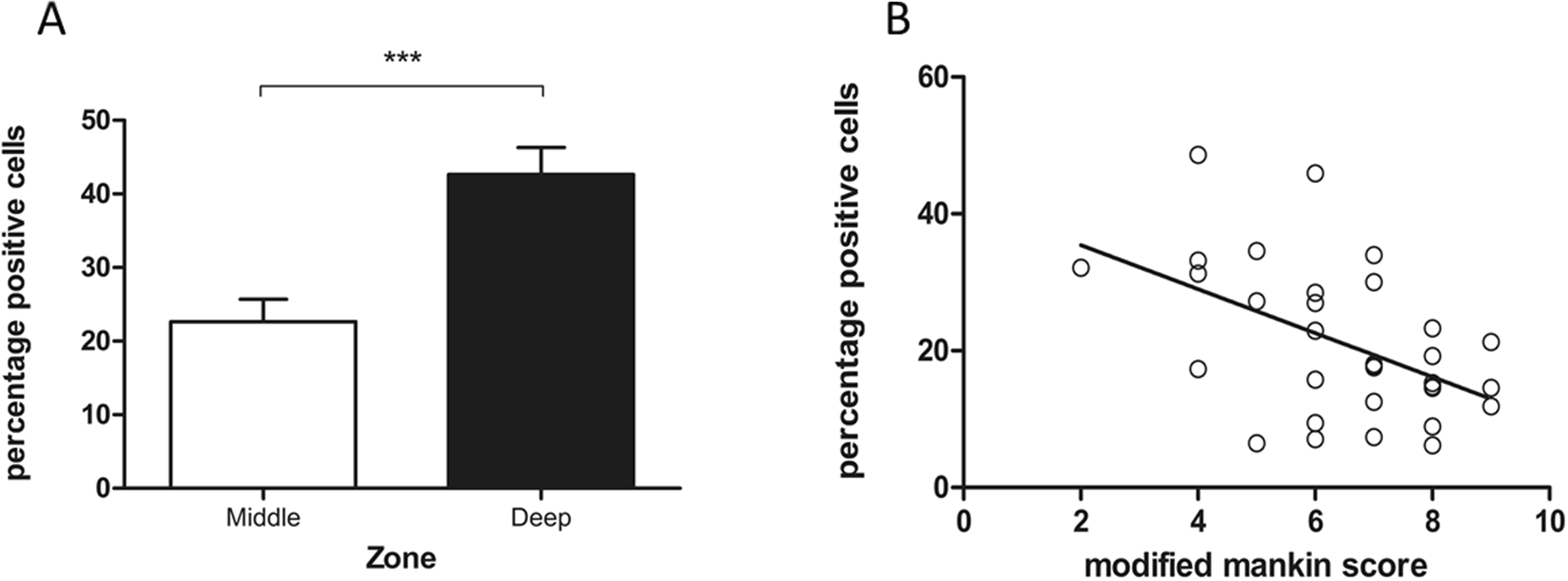

Supplement: Supplementary file 2 — Authors’ original file for figure 2 [file 12891_2014_2379_MOESM2_ESM.tif]

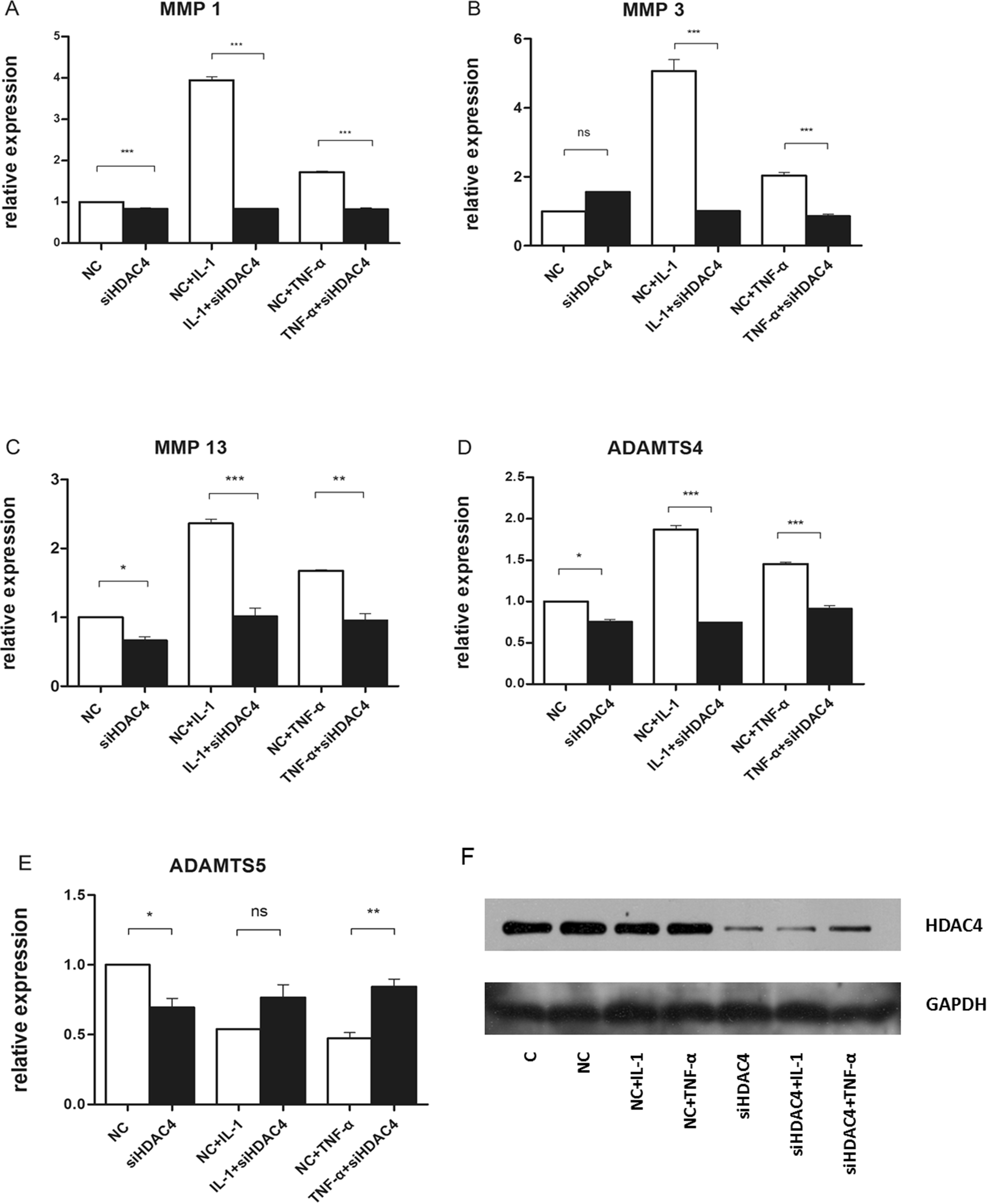

Supplement: Supplementary file 3 — Authors’ original file for figure 3 [file 12891_2014_2379_MOESM3_ESM.tif]

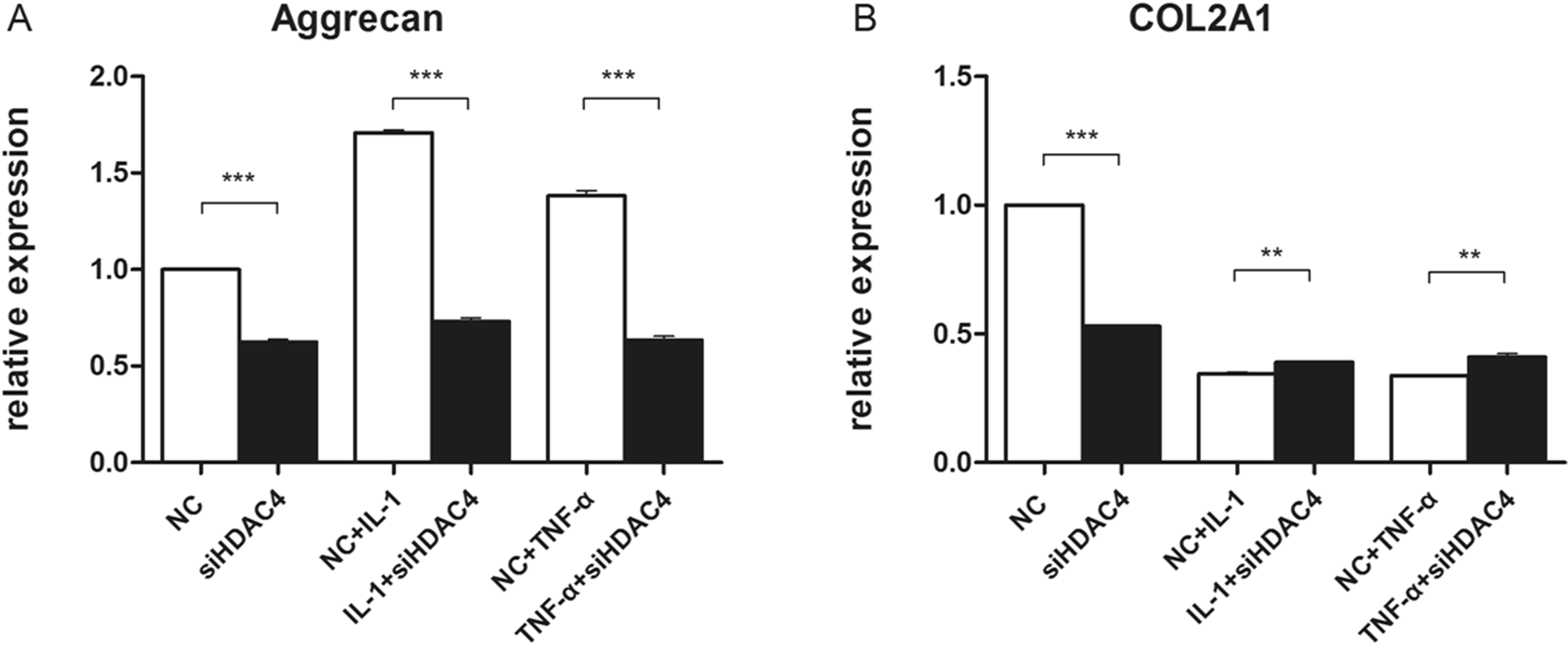

Supplement: Supplementary file 4 — Authors’ original file for figure 4 [file 12891_2014_2379_MOESM4_ESM.tif]
